# Familial Clustering of Unexplained Transient Respiratory Distress in 12 Newborns from Three Unrelated Families Suggests an Autosomal-Recessive Inheritance

**DOI:** 10.1100/tsw.2007.252

**Published:** 2007-09-28

**Authors:** Andrea Guala, Paola Carrera, Guido Pastore, Marco Somaschini, Gina Ancora, Giacomo Faldella, Paolo De Filippi, Federica Ferrero, Roberta Guarino, Cesare Danesino

**Affiliations:** ^1^ SOC Pediatria Osp., Castelli Verbania, Italy; ^2^ Laboratorio di Biologia Molecolare Clinica, IRCCS Osp. S. Raffaele Milano, Italy; ^3^ SOC Pediatria, Osp. S.Andrea, Vercelli, Italy; ^4^ SOC Neonatologia e Patologia Neonatale, Osp. Bolognini, Seriate, Italy; ^5^ Istituto di Neonatologia, Università di Bologna, Italy; ^6^ Genetica Medica, Università di Pavia e IRCCS S. Matteo, Pavia, Italy; ^7^ Clinica Pediatrica, Università del Piemonte Orientale, Novara, Italy

**Keywords:** respiratory distress, newborn, familial clustering

## Abstract

We report on 12 near-term babies from three families in which an unexplained transient respiratory distress was observed. No known risk factor was present in any family and no sequelae were recorded at follow-up. The most common causes of respiratory distress at birth are Neonatal Respiratory Distress Syndrome (NRD) and Transient Tachypnea of the Newborn (TTN), and their cumulative incidence is estimated to be about 2%. Genetic factors have been identified in NRD (surfactant genes) or suggested for TTN (genes affecting lung liquid clearance). Survivors from NRD may develop clinically relevant sequelae, while TTN does not cause any problem later in life. Our cases do not immediately fit NRD or TTN, while familial recurrence suggests the existence of a previously unreported subgroup on patients with respiratory distress for which autosomal-recessive inheritance is likely.

## INTRODUCTION

Neonatal Respiratory Distress Syndrome (NRD) is usually found in preterm babies, and is one of the major causes of morbidity and mortality. Its incidence in Italy is estimated to be 1.15%[1]. Evidence for the importance of genetic factors in determining the susceptibility and severity of NRDS has been suggested by twin studies; it occurred more frequently in both twins when the twins were monozygotic (12/18; 67%) than when they were dizygotic (18/62; 29%)[2]. So far, linkage studies have not helped in identifying genetic susceptibilities to NRDS because large families with multiple affected individuals are not available, given that many affected infants died in infancy. The candidate gene approach, however,

*A.G. and P.C. equally contributed to this work.
**Corresponding author.
©2007 with author.
Published by TheScientificWorld; www.thescientificworld.com demonstrated the causal role of a quantitative deficit due to mutated pulmonary surfactant proteins[3,4]. Survivors frequently develop broncopulmonary dysplasia, chronic pulmonary disease, or asthma in childhood[5]. A review on the synthesis, storage, secretion, recycling, and catabolism of alveolar surfactant was recently published by Hallman[6].

Transient Tachypnea of the Newborn (TTN) is usually a benign, self-limiting respiratory disease observed in the immediate postnatal period. Risk factors include cesarean delivery, maternal diabetes, and twin pregnancy. A delayed clearance of fetal lung liquid at birth, which causes an altered respiratory function that transiently may compromise gas exchanges, is the proposed pathogenetic mechanism[7]. The involvement of genes affecting lung liquid clearance has been hypothesized[8], while no relevance of surfactant B gene (SFTP3) in the etiology of TTN was demonstrated[9]. Its incidence in Italy is estimated to be 0.93%[1] and, to the best of our knowledge, no familial cases have been reported.

We report on three families (without known risk factors) in which 12 near-term babies presented with a neonatal respiratory disease, clearly different from the well-known severe NRDS of the preterm babies, but also from TTN. A long-time follow-up failed to disclose any sequelae. Recurrence of the disease in these families suggests the existence of a subgroup of cases for which an autosomal-recessive inheritance is likely.

## CASE REPORT

Family 1 (Fig. 1) — Respiratory distress (rd) occurred in two branches of the family. Three of the grandparents (II,1,2, and 3) come from the same small village (less than 500 inhabitants) and share the same family name, even if consanguinity is not formally proven between II,1 and 2. II,4 is apparently unrelated, but comes from a small nearby village. Anamnestic records show that none of their children (III,2,3,5,7,9, and 11) presented with rd at birth.

All parents of the affected children (III,3 and 4) (III,10 and 11) (III,12 and 13) come from the same village or from closely placed small villages located in the same Alpine valley, in Northern Italy. A synopsis of clinical data of affected members of the family (IV,2,4,8,9, and 11) is entered in Table 1.

Family 2 (Fig. 1) — Parents are unrelated and their ancestors come from geographically different areas from Northern Italy. Clinical data of cases II,4,5,6,7, and 8 are entered in Table 1.

Family 3 (Fig. 1) — Parents are unrelated, but their ancestors (6/8 grandparents) come from the same geographical region, and two of the family names (II,2 and 3) are common in the villages of origin of the family. Clinical data of cases III,2 and 3 are entered in Table 1.

In all cases, evidence of respiratory distress was recorded immediately after birth, and improvement or recovery was observed only after at least 2 days of therapy.

All the affected subjects were tested for mutations within the SFTP3 gene; no mutation was found, but only DNA variants already described as common polymorphisms.

## DISCUSSION

NRD is the most frequent respiratory cause of death in infants below 1 year of age. Prematurity is the most common cause of NRDS and acts through a maturation deficiency of surfactant. Clusters of NRDS cases within the same family and differences between ethnic groups suggest the relevance of genetic causes in the pathogenesis of the disease[2,10,11].

The genes coding for surfactant proteins A, B, C, and D have been widely studied, and their association with autosomal-recessive NRDS or other respiratory disorders have been reviewed[12]. Genetically determined forms of NRDS are often lethal at birth or show relevant sequelae in survivors. Mutations in SFTP3 (surfactant protein B), even if quite rare, cause the most common form of genetically determined NRDS. Zimmermann et al.[13] report that some mutations may be associated to transient surfactant protein B deficiency. A case of an at-term newborn characterized by “severe respiratory failure and transient surfactant protein B deficiency" showed clinically relevant sequelae. At 3 years of age, the patient required supplemental oxygen because of chronic lung disease[14] .

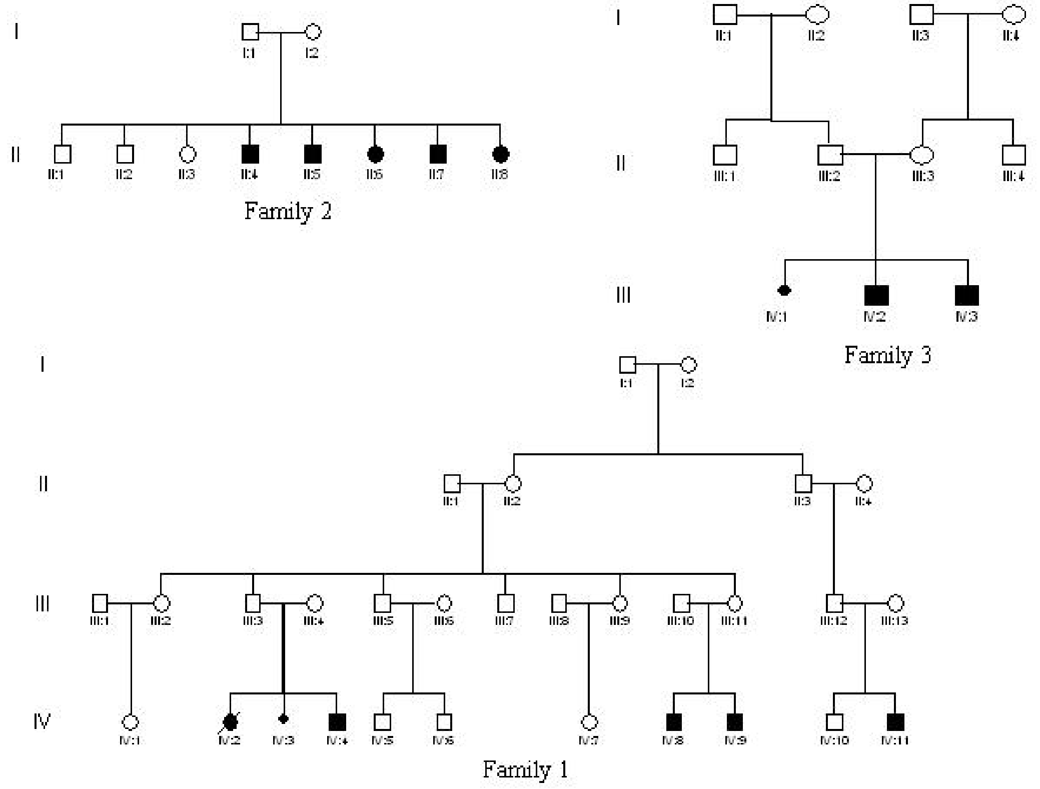

**FIGURE 1**

NRD has been observed also in near-term babies where pathogenetic factors unrelated to prematurity need to be considered. These may include factors relevant for lung maturation as steroids, thyroid hormones, β-agonists, and γ-interferon[15]. Tredano et al.[16] collected a large series of at-term newborns affected with lethal or very severe unexplained respiratory distress in which most of survivors showed severe sequelae at follow-up. Genetic causes were likely to be present, as many families showed consanguinity or recurrence of the disorder in subsequent children. In fact, mutations in the surfactant B protein were identified in 9/40 index cases. In one of the reported families (URD29, affected brother and sister from unrelated parents), the respiratory distress, at variance to all other families, was transient and no mutations in the SFTPB gene were found. Apart from this single family, we did not find other families in the literature with similar mild and transient expression of the disease.

TTN is a benign, common respiratory disorder occurring in the immediate neonatal period, for which no data on familial occurrence are available. In a recent report, Agrawal and coworkers[17] stress the need for an improved classification of respiratory disorders of the newborn, and suggest a new category ("transient respiratory insufficiency of the newborn") for babies ventilated briefly, but without demonstrable surfactant deficiency or infections. They also state that this category includes infants with many different etiologies. No data are included as to a possible genetic origin of this condition.

The families we report showed a clinical picture clearly different from NRD, as there was no specific radiological pattern and no increasing oxygen dependence during the first 24 h, which are the diagnostic criteria reported in Dani et al.[1].

Similarly, the clinical picture was more severe than expected for TTN as mechanical ventilation was needed for several days (see Table 1) and the clinical picture did not improve within 3–6 h as requested in the diagnostic criteria[1].

**TABLE 1**
**Synopsis of Main Clinical Data**

| Case | Year of Birth | Gestation (weeks) | Birth Weight (g) | Apgar | Symptoms | Oxygenation Index | Chest X-Ray | Follow-Up |
|---|---|---|---|---|---|---|---|---|
| **Family 1** | | | | | | | | |
| IV,2 | 1983 | 40* | 5000 | 10/10 | Cyanosis, tachypnea; mechanical ventilation | 5 | Not available | Died because of heart malformation |
| IV,4 | 1986 | 37* | 3600 | 3/5 | Cyanosis, tachypnea; $O_2$ therapy for 2 days | — | Normal | ND at 20 years of age |
| IV,8 | 1999 | 38* | 3100 | 8/8 | Cyanosis, tachypnea; mechanical ventilation for 2 days | 6.2 | Diffuse parahilar broncovascular congestive radiating pattern | ND at 7 years of age |
| IV,9 | 2001 | 38* | 3100 | 8/9 | Cyanosis, tachypnea; $O_2$ therapy for 3 days | — | Bilateral increase of the bronchovascular pattern | ND at 5 years of age |
| IV,11 | 1996 | 37* | 3200 | 4/5 | Cyanosis, tachypnea; mechanical ventilation for 3 days | 4,6 | Diffuse parahilar broncovascular congestive radiating pattern and a small right pneumothorax | ND at10 years of age |
| **Family 2** | | | | | | | | |
| II,4 | 1981 | 37* | 3150 | 6/7 | Cyanosis, tachypnea; mechanical ventilation for 6 days | 11 | Severe interstitial congestive broncoalveolar pattern | ND at 25 years of age |
| II,5 | 1982 | 36* | 3500 | 9/10 | Cyanosis, tachypnea; mechanical ventilation for 9 days | 4,2 | Typical findings of retained fluid syndrome | ND at 24 years of age |
| II,6 | 1983 | 37* | 3950 | 9/9 | Cyanosis, tachypnea; C-PAP ventilation for 2 days | — | Slight bilateral lung bases haziness | ND at 23 years of age |
| II,7 | 1986 | 36* | 5400 | 8/9 | Cyanosis, tachypnea; C-PAP ventilation for 3 days | — | Diffuse parahilar bronchovascular congestive radiating pattern | ND at 20 years of age |
| II,8 | 1988 | 36* | 4950 | 8/9 | Cyanosis, tachypnea; $O_2$ therapy for 3 days | — | Slight bilateral lung bases haziness | ND at 18 years of age |
| **Family 3** | | | | | | | | |
| III,2 | 1999 | 35** | 2640 | 5/5 | No spontaneous breathing; mechanical ventilation for 3 days | 13 | Fully normal | ND at 7 years of age |
| III,3 | 2003 | 35** | 2720 | 5/5 | No spontaneous breathing; C-PAP was continued for 7 days | — | Typical findings of retained fluid syndrome | ND at 3 years of age |

* Uncomplicated vaginal delivery.

** Caesarean section because of dynamic dystocia.

Oxygenation index: $FiO_2 \times$ MAP (Mean Airway Pressure) $\times 100/PaO_2$. An Oxygenation Index $\geq$40 in neonates for a minimum of 2–4 h identifies a mortality risk of at least 80%.

ND: normal development.

Moreover for our cases, we consistently observed familial recurrence; full- or near-term vaginal delivery; absence of predisposing factors, such as maternal diabetes or asthma; severe presentation of respiratory distress (requiring mechanical ventilation in 6/9 cases) with transient course of the disease that resolved without any sequelae. It should be stressed that our follow-up ranges from 3 to 25 years, and that older patients consistently show a fully normal respiratory function.

None of our cases showed wheezing disorder in childhood, which has recently reported by Liem et al.[18] to be observed at increased frequency in infants affected with TTN at birth.

We collected our data retrospectively, and no measurement of surfactant protein was done, as when the patients were born, delivery occurred near-term, and molecular analysis of SFTP3 (OMIM 178640) (tested because of the possible association with transient surfactant protein B deficiencies) failed to identify any pathogenetic mutation in all families.

The structure of the three pedigrees is in agreement with the hypothesis of a recessive trait: consanguinity in family 1 is very likely even if not proven, and in family 3 is possible; both males and females are affected. Of course, the hypothesis of an X-linked recessive trait cannot be excluded in family 3. The ratios affected/unaffected and male/female are slightly different from expected values, but the size of the sample is too small for statistical analysis and speculation.

A genetic cause for respiratory distress in our families is very likely, and it may involve proteins whose action is related to surfactant proteins or affecting lung liquid clearance[8]. DNA samples from these families are available for further studies.
